# Mapping of Research in the Field of Forest Therapy-Related Issues: A Bibliometric Analysis for 2007–2021

**DOI:** 10.3389/fpsyg.2022.930713

**Published:** 2022-07-11

**Authors:** Xin Wang, Xiang-Fei Gong, Ke-Xin Xiong, De-Sheng Guo, Li-Jun Liu, Chia-Min Lin, Wei-Yin Chang

**Affiliations:** ^1^Laboratory of Environmental Education and Forest Therapy, College of Forestry, Fujian Agriculture and Forestry University, Fuzhou, China; ^2^Forest Therapy Branch, Chinese Society of Forestry, Beijing, China; ^3^Taiwan Forest Therapy Society, Taipei, China

**Keywords:** forest therapy, green spaces, bibliometric analysis, intellectual development, human well-being

## Abstract

Exposure to forest environments promotes human health. The number of relevant studies in this area has increased rapidly. However, an overall review of relevant analyses from the perspectives of bibliometrics and visualization is lacking. A scientometric analysis of 2,545 publications from 2007 to 2021 *via* the Web of Science database was conducted to identify the knowledge structure and frontiers objectively. The publications were subsequently analyzed in terms of the distribution of journals and countries, citation bursts, major subject areas, and evolutionary stages. The findings showed that the knowledge foundation of forest therapy was multidisciplinary with most published in the fields of environmental sciences and ecology but lacking input from social disciplines. The research hotspots evolved from the early focus on individual benefits obtained from nature to increasing attention on human well-being at the social-ecological scale. More rigorous experiments with strict randomized controlled trials and blinding are needed to accommodate the trend of forest therapy toward non-pharmacological treatments. According to Shneider’s four-stage theory, forest therapy research is in the third stage of the scientific research process. More future studies utilizing novel technologies and decision-making frameworks to solve practical issues are needed for introducing health into policies and promoting human well-being.

## Introduction

Health plays a very important role in people’s lives and is of great significance to social stability and economic development, especially in the context of the post-COVID-19 era (Курюкин, 2020; Hermans et al., 2021; Ishikawa et al., 2021). Scientific data show that adults experience an average of four unhealthy symptoms every 14 days (Jadad and O’Grady, 2008; Leonardi, 2018). Along with the rising level of health awareness, people are investing heavily in health research and development (Tang and Li, 2022). The 2021 annual report from the World Health Organization showed that over the past two decades, global spending on health has more than doubled in real terms (Global Health Expenditure Database [Www Document], n.d.), and this has paid off in a big way (Jamison et al., 2013), with people living longer while being healthier than ever before (Obrecht, 2015).

Contact with nature is one of the most convenient and effective ways to improve health. It spans traditional passive healing through living in a natural environment to active use of nature by returning from the city to the forest for healing and relaxation (Park et al., 2007; Song C. et al., 2016). An alternative health promotion approach is forest therapy. It is a complementary and alternative treatment method, with the forest environment to be the foundation and the preventive medicine to be the core (Li, 2013; Zhang et al., 2020; Stier-Jarmer et al., 2021). Improving physical and mental health and preventing disease is the end goal of forest therapy by carrying out a set of activities in a specific forest environment to make people consciously use all five senses, such as forest retreats, forest walks, and others (Lee et al., 2017; Zhang et al., 2020; Antonelli et al., 2021; Stier-Jarmer et al., 2021). Forest therapy was first proposed by the Japanese Ministry of Agriculture, Forestry, and Fisheries under the term “forest bathing” in 1982 (Antonelli et al., 2021), and has subsequently gained attention around the world. For example, in South Korea, the Forestry Agency launched the Human Health Forest Project in 2007 (Shin et al., 2009). In China, four national departments jointly issued Opinions on Promoting the Development of the Forest Health and Wellness Industry to advance the development of this therapy in 2019 (Zhang et al., 2020). The concept of forest therapy has also been developed in Europe in different countries, such as “Kneipp Therapy” in Germany, “Green Steps” in Sweden, “Power Trail at Ikaalinen Spa” in Finland, and “Nacadia Therapy Forest Garden” in Denmark (Locher and Pforr, 2014; Stier-Jarmer et al., 2021). Available studies have shown that forest therapy has a positive association with health. Physiologically, the forest environment has been found to have helpful effects on physical relaxation and immune system strength (Park et al., 2007; Li et al., 2010; Tsunetsugu et al., 2013). Psychologically, accumulated evidence indicates that forest therapy can promote a positive mood by making subjects feel more relaxed, comfortable, and energetic with less tension, anxiety, and fatigue (Park et al., 2011; Tsunetsugu et al., 2013; Lee et al., 2014; Ochiai et al., 2015b; Liu Q. et al., 2021).

The relevant literature shows a rapidly increasing tendency. Through a topic search of the term “forest therapy”, the Web of Science (WoS) returned 185 records from the past 5 years. When records relevant to forest therapy but without the term “forest therapy” were precisely searched, the number of references increased to 10,278. It is essential to keep pace with the rapidly growing literature. New findings are often appearing in a variety of different research areas, and may change collective knowledge radically (Chen, 2012). Faced with such a large body of literature, it has become important for researchers to grasp the structure of disciplinary knowledge and the frontiers of development in a relatively short period of time.

Bibliometrics is increasingly being used to map the structure and development of scientific fields and disciplines. For example, this method has been widely used in the fields of geographic information system (GIS) (Wei et al., 2015), acupuncture (Liang et al., 2017), hospitality (Xinjian Li et al., 2017), and others. Furthermore, bibliometrics is also used extensively in forest-related research (Bullock and Lawler, 2015). Relevant research objects include the status of *Picea* (Duan et al., 2020), forest carbon sequestration (Huang et al., 2020), the forest bioeconomy (Paletto et al., 2020), and forest ecosystem services (Aznar-Sanchez et al., 2018).

Integrating previous research findings is one of the most significant tasks for the advancement of a particular line of research (Zupic and Èater, 2015). A number of literature reviews on forest therapy have been completed in the past few years (Kamioka et al., 2012; Lee et al., 2017; Meyer-Schulz and Buerger-Arndt, 2019; Rajoo et al., 2020; Stier-Jarmer et al., 2021). However, most previous research has been expert-dependent on unavoidable subjective and individual preferences (Fang et al., 2018). Quantitative bibliometric analyses focusing on forest therapy research at the global level are lacking.

To present an overall and in-depth review of forest therapy and related issues objectively, we provide a literature review utilizing a scientometric analysis. Specifically, our aims were as follows:

(1) To identify research focuses, emerging trends, and the evolution tendency of forest therapy-related fields.
(2) To distinguish the key knowledge groups and identify the current gaps and future directions.
(3) To detect emerging topics and present an overview of the forest therapy situation from a cross-disciplinary perspective.

## Materials and Methods

### Data Collection

The data used in this study were filtered from the Web of Science Core Collection (WoSCC) database on 17 November 2021. The WoS is one of the most widely used multidisciplinary citation indexing databases and is an ideal data source for bibliometrics (Liu et al., 2015; Fang et al., 2018; Birkle et al., 2020), which also has good compatibility with CiteSpace (Zhang et al., 2021). In this study, we selected WoSCC to obtain more comprehensive metadata (i.e., title, author, publication information, abstract, and references) related to each publication (Miao et al., 2017).

In this study, all titles, abstracts, and keywords were searched using the following terms: “Forest therapy,” “Forest bathing,” “Shinrin yoku,” “Nature therapy,” and “Forest medicine.” The search language was English and all of the above terms were combined with the Boolean operator OR. These terms were selected from a careful analysis of supporting literature, where the terms mentioned above were usually used in conjunction with each other in recent reviews of the forest therapy scholarly literature (Hansen et al., 2017; Antonelli et al., 2021). Based on the topic research, the core dataset was reduced to 290 original articles by manually and quickly reviewing the titles and abstracts of the literature and sifting out the less representative articles (Chen, 2017). The result of the topic research constitutes the core dataset. Then we conducted the construction of the expanded dataset to explore more issues and emerging trends related to forest therapy, based on the assumption that if an article cites at least one article in the core dataset, it may be thematically relevant to the research topic (Chen, 2017). This method of citation expansion is derived from the principle of citation index proposed by Garfield and Sher (1963), which has been adopted by many bibliometric studies to maximize the recall and the precision simultaneously with the least effort (Chen et al., 2012, 2014; Kim and Chen, 2015; Li et al., 2017). The bibliometric analysis was based on both datasets of 2,545 articles, including 2,123 (83.4%) original articles and 422 (16.6%) reviews.

### Bibliometric Analysis

**FIGURE 1:**
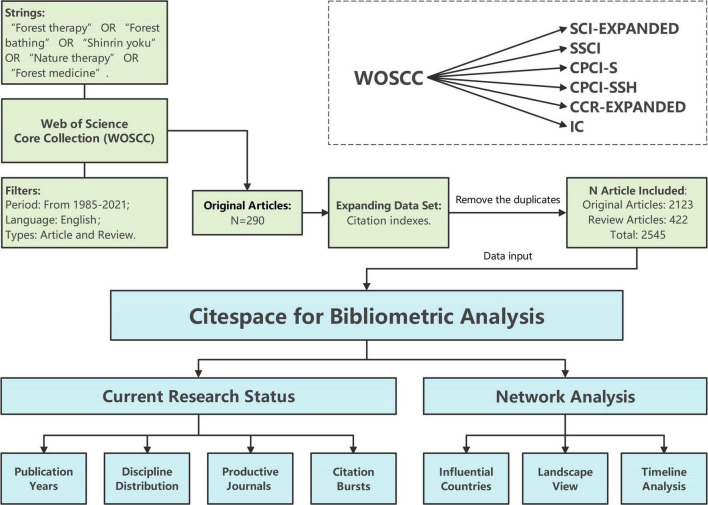
Methodology steps used in the bibliometric analysis developed for forest therapy.

In this manuscript, CiteSpace (version 5.8.R3) was used to analyze and visualize the forest therapy research area. According to the definition presented in CiteSpace, nodes and lines represent different references and citation relationships in the visualization knowledge maps, respectively. The cloors of nodes and links represent different years (Liang et al., 2017). The difference in citation frequency is reflected by the size of nodes, and the thickness of links indicates the co-citation frequency (Chen et al., 2012). Additionally, references can be identified and classified into different clusters based on the co-citation relationships, which can help recognize diverse research subtopics across all manuscripts (Chen et al., 2012; Shi and Liu, 2019; Fang et al., 2020). The procedure of the data collection and bibliometric analysis is shown in Figure 1.

## Results

### Publication Year

**FIGURE 2:**
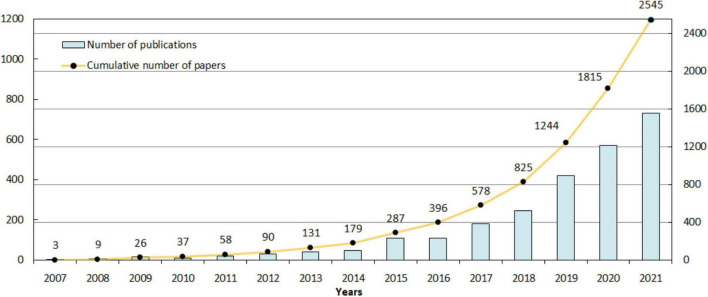
Annual growth in the number of publications in our research area.

Figure 2 shows that examined literature in this field was published between 2007 and 2021. The first article was published in 2007, and since then, the number of publications has been on the rise. From 2007 to 2014, the volume of articles grew slowly. The number of articles published between 2015 and 2019 reached nearly quadruple of the previous period. Since 2020, the publications have met a massive increase, which may be related to the COVID-19 pandemic. It should be noted that, as the time of data collection is 17 November 2021, the data for 2021 would be incomplete.

### Distribution of Journals

#### Disciplines Distribution

The knowledge map of discipline distribution shows that the research on forest therapy crossed numerous disciplines (Supplementary Figure 1). The fields of environmental sciences and ecology have the largest number of studies. There are also many articles in the areas of occupational health, urban studies, forestry, and psychology.

#### Influential Journals

The top 10 influential journals in the forest therapy field are listed in Table 1. The top five journals in terms of the number of publications in this area are *International Journal of Environmental Research and Public Health* (13.3%), *Urban Forestry and Urban Greening* (5.6%), S*ustainability* (3.9%), *Landscape and Urban Planning* (3.18%), and *Frontiers in Psychology* (3.1%).

**TABLE 1:** The top 10 journals containing references to forest therapy research.

Rank	Journal	TCb	N/2545 (%)	IFa	EF	AI
1	*International Journal of Environmental Research and Public Health*	339	13.30	2.948	0.023	0.7
2	*Urban Forestry and Urban Greening*	142	5.58	5.356	0.003	0.6
3	*Sustainability*	101	3.96	3.473	0.007	0.4
4	*Landscape and Urban Planning*	81	3.18	7.96	0.013	1.0
5	*Frontiers in Psychology*	74	2.91	3.618	0.047	1.0
6	*PLOS One*	52	2.04	3.788	1.814	1.1
7	*Journal of Environmental Psychology*	46	1.81	7.871	0.006	1.3
8	*Environmental Research*	45	1.77	6.824	0.014	1.2
9	*Forests*	45	1.77	2.453	0.003	0.5
10	*Health and Place*	43	1.69	5.145	0.013	1.1

*TCb, the total number of citations in the journal; N, number of publications in the corresponding journal; IFa, Five-year impact factor, impact factor data from the 2020 edition of Journal Citation Reports^®^ in Web of Science; EF, Eigenfactor^®^ score; AI, Article Influence^®^ score.*

### Most Influential Countries and Regions

Table 2 lists the top 10 influential countries and regions based on the contributions. Higher values of betweenness centrality indicate stronger communication and cooperation in the collaboration network (Abbasi, 2013). The United States of America, China, and England have the highest nubmer of publications. The centralities of The United States of America and England also rank in the top three while the centrality of China ranks 30. Only three countries are in the Asia-Pacific region (China, Japan, and South Korea) while others are located in North America and Europe.

**TABLE 2:** The top 10 countries and regions in terms of publications.

Countries/Regions	Number of manuscripts	Centralities (Rank)	Year
United States	586	0.18 (2)	2007
China	447	0.01 (30)	2009
England	322	0.31 (1)	2009
Australia	219	0.17 (3)	2008
Japan	206	0.03 (20)	2008
South Korea	162	0.03 (21)	2010
Germany	149	0.05 (11)	2008
Spain	131	0.13 (4)	2012
Canada	130	0.07 (5)	2012
Sweden	121	0.04 (15)	2010

### Author Cooperation Network

**FIGURE 3:**
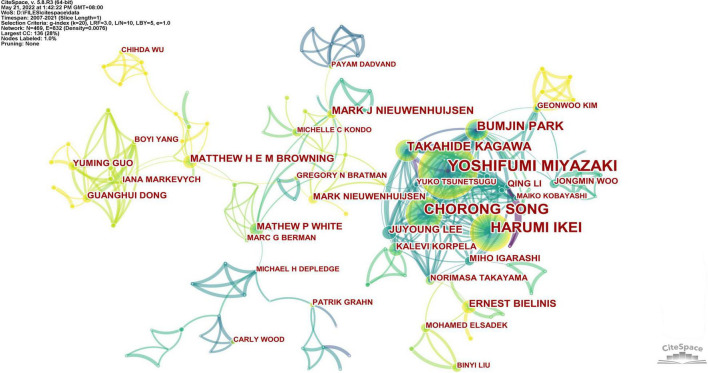
The collaboration network of authors that published studies on forest therapy.

The author’s collaboration network is demonstrated in Figure 3, including a total of 469 nodes and 832 edges. As can be seen in the figure, the cooperation map includes a part of relatively small independent networks that have not formed a scale, as well as several large cooperation networks. For example, one of the most typical groups is established among Miyazaki, Ikei, Song, Park, and Kagawa to explore the restorative effects of the forest therapy on people (Kobayashi et al., 2018), and they are also among the top five authors with the greatest number of manuscripts in this area. Another productive collaborative network is established by Ojala, White, Browning, Bratman, and others based on the research interests related to the environment and health (Olvera-Alvarez et al., 2021; White et al., 2021). Their centralities are all in the top five, higher values of which indicate stronger communication and cooperation in networks (Abbasi, 2013).

### High-Frequency Keyword Analysis

#### Keyword Co-occurrence Analysis

**FIGURE 4:**
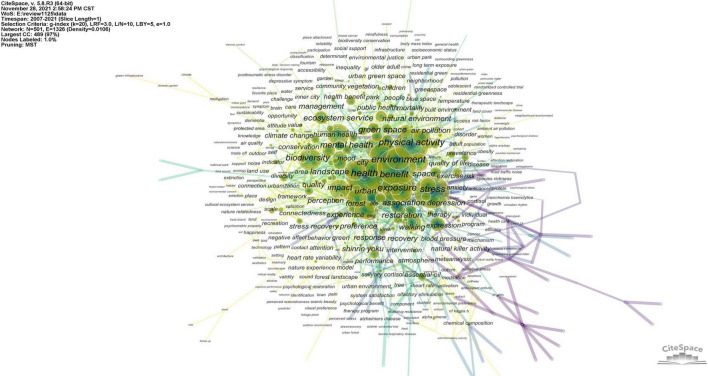
Keyword co-occurrence visualization network.

The keywords of one manuscript can reflect the critical content of the research. The network map of keyword co-occurrence can be used to explore the frequency and relationship of keywords. Figure 4 represents the results of keyword co-occurring analysis with the data extracted from the 2,545 retrieved articles of a core dataset and expanded dataset. The sizes and links of the nodes represent the occurrence frequency and the link strength of keywords, respectively. The keywords can be roughly divided into two types, one group is about the human state, such as health (460), benefit (337), physical activity (331), stress (316), and so on, while another group is mainly concerning the space, such as environment (395), exposure (285), green space (213), and forest (125).

#### Keyword Citation Burst Analysis

**FIGURE 5:**
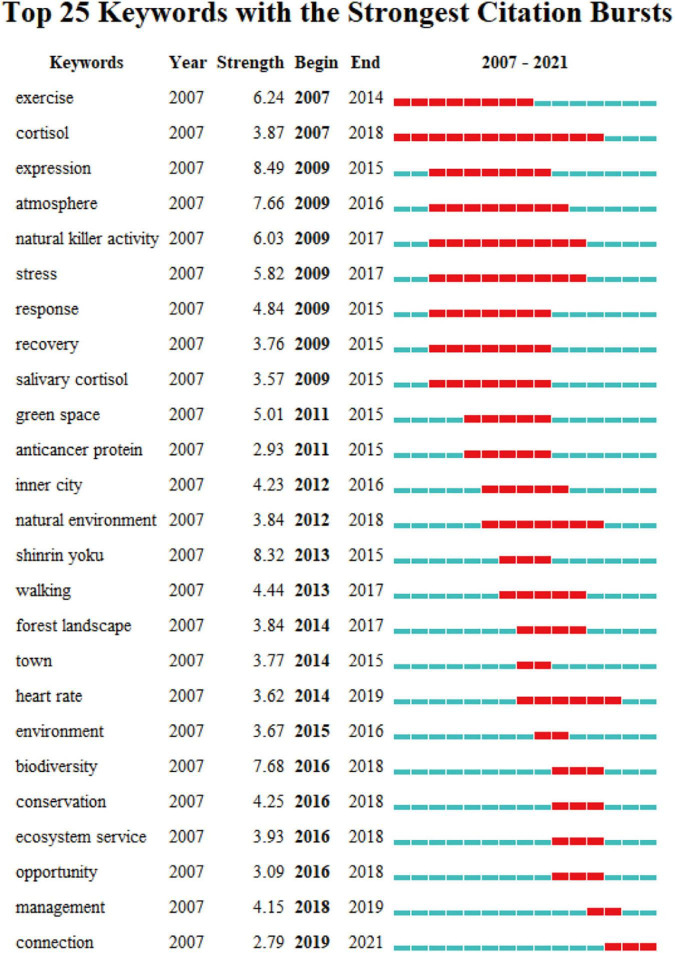
The keywords with the strongest citation bursts in publications on forest therapy research.

Figure 5 shows the burst strength and time span of the top 25 keywords in the past 15 years. Each blue bar in the figure represents a year. The blue bar turning red means that the keyword exploded, and the corresponding keyword is the research hotspot of that year. The majority of keywords that were first used before 2012 were related to physical indicators, such as “exercise,” “natural killer activity,” “salivary cortisol,” and “anticancer protein.” Since 2012, some new research considered various environments with the appearance of “inner city,” “natural environment,” “forest landscape,” and “town.” Research topics after 2016 were focused on the rethinking of the relationships between humans and nature, reflected by the burst of “biodiversity,” “conservation,” “ecosystem service,” “management,” and the newest frequently cited keyword “connection.”

### Landscape View

**FIGURE 6:**
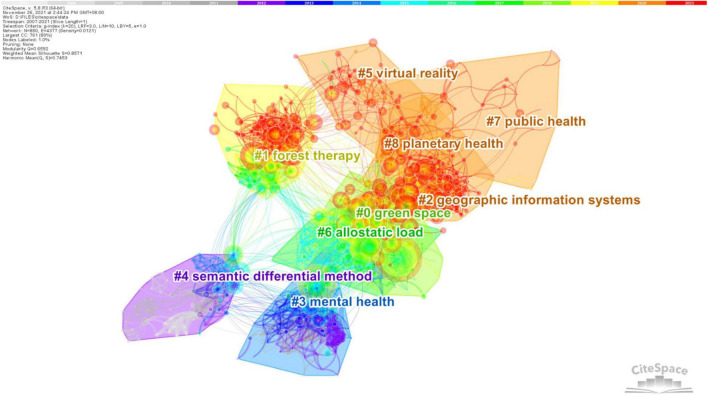
A landscape view of the main clusters.

Cluster analysis can reflect the knowledge structure and research focus of a subject according to the citation relationship (Song J. et al., 2016). We used the g-index (k = 20) as the selection criterion and the generated network consisted of 49 clusters from 2,545 references (Figure 6). The modularity of the network was 0.6592 and the average silhouette score was 0.8571, suggesting a clear definition of co-citation and high-level homogeneity of clusters (Chen, 2017). Clusters were numbered according to the size, with the largest one being Cluster #0.

### Cluster Analysis

**FIGURE 7:**
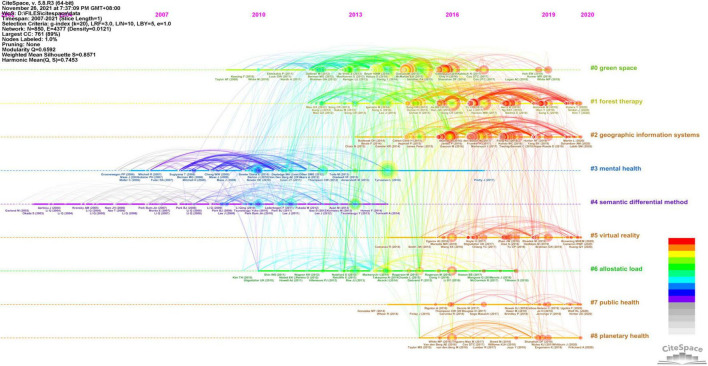
A timeline visualization of the main clusters.

The timeline visualization generated in CiteSpace illustrates the development of the above clusters along horizontal timelines (Figure 7). Clusters are displayed from left to right with the largest cluster shown at the top. A large node indicates a reference with a high citation frequency and hence a strong analysis value. The top three most cited articles every year are listed below the corresponding node. As the largest three clusters include 15.5% of the references from the entire network, we mainly focused on the largest three clusters according to the interpretation of Chen (2017).

#### Cluster #0

The largest cluster, Cluster #0, is labeled as green space and contained 152 members. The core idea of the top 10 most cited references (Supplementary Table 1) is that, compared to spending time in an urban environment, contact with nature can provide benefits to both physiological and psychological health.

**FIGURE 8:**
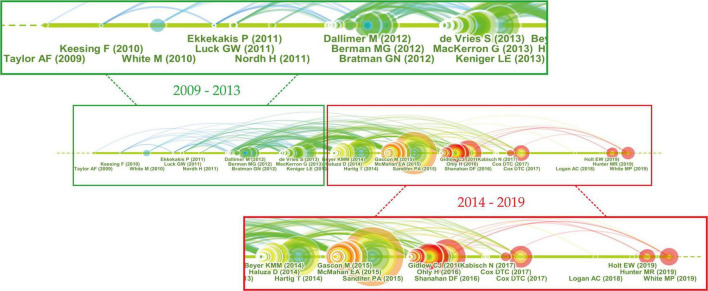
High-impact publications in Cluster #0.

From the timeline visualization of the “green space” cluster (Figure 8), we divided development in this area into two periods. The first period was from 2009 to 2013. This was a relatively uneventful period, containing no references with large citation tree rings or red citation bursts. The most frequently cited manuscript in this period was written by Keniger et al. (2013), in which three categories of interaction with nature and six types of benefits were identified.

The second period was from 2014 to 2019 with prominent references in terms of citation counts or bursts. These high-impact contributions can be approximately divided into two types: literature reviews and theory applications. Five reviews analyzed the relationship between nature and health from different points of view. Hartig et al. (2014) summarized the benefit mechanisms of nature exposure and human health, involving four aspects of air quality, physical activity, social cohesion, and stress reduction. Evidence on the positive association was found between biodiversity and production of ecosystem services (Sandifer et al., 2015), nature contact and emotional well-being improvements (McMahan and Estes, 2015), nature experience and attention restoration theory (Ohly et al., 2016), while the evidence of surrounding greenness to mental health was found limited (Gascon et al., 2015).

Over time, researchers have paid more and more attention to the application of theories relating to green space and have attempted to answer the question proposed by the abovementioned researchers (Keniger et al., 2013; Gascon et al., 2015; Sandifer et al., 2015) to obtain the best health outcomes, how much engagement with nature do people need, and which characteristics of nature are most important? Most studies found that 30 mins contact with nature had positive effects on reducing depression, high blood pressure (Gidlow et al., 2016), and stress (Shanahan et al., 2016). The most efficient benefits of nature-based stress reduction happened in durations between 20 and 30 mins (Hunter et al., 2019). Levels of self-reported health and subjective well-being were highest when the time spent in nature per week was between 200 and 300 mins with no further gain after that (White et al., 2019). Besides, when the vegetation cover around living neighborhoods was more than a threshold of 20%, the chance of getting depression and anxiety was reduced significantly (Cox et al., 2017).

Recurring themes in the citing articles reflect the interrelationship between the intellectual base and the research fronts (Chen, 2017). As shown in Supplementary Table 2, the top 10 citing articles in Cluster #0 paid constant attention to the relationship between nature and health (2, 4, 7, 8, and 9). Moreover, since mental illness accounts for a large proportion of suffering in all regions of the world (Steel et al., 2014), there have been studies that focused on mental health from different perspectives, such as ecosystem services and biodiversity conservation (1, 6).

#### Cluster #1

The second-largest cluster was Cluster #1, labeled forest therapy. This cluster contained 123 references spanning from 2012 to 2020. From the summary of the most cited references (Supplementary Table 1), we found restorative effects of forest experience in a relatively small sample size to be the main study interest in this cluster.

**FIGURE 9:**
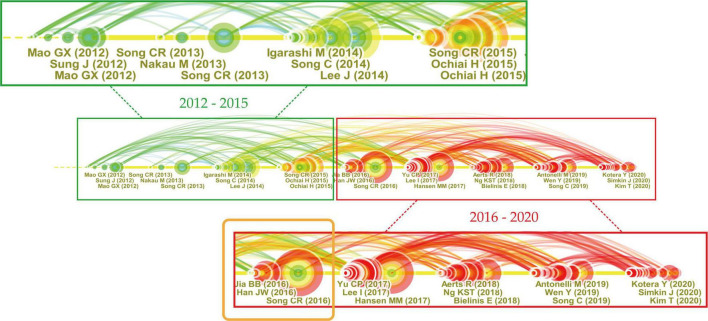
High-impact publications in Cluster #1.

Figure 9 showed how study spotlights evolved. Most of the highly cited articles from 2012 to 2015 were experimental studies on different groups, offering referred experimental methods for later research. The target beneficiaries varied from healthy young adult males (Song et al., 2013, 2015; Lee et al., 2014) and middle-aged females (Ochiai et al., 2015a) to middle-aged males with high-normal blood pressure (Ochiai et al., 2015b) and elderly patients with essential hypertension (Mao et al., 2012). The measurements differed according to research aims. Blood pressure was the most commonly used physiological parameter (Lee et al., 2014; Ochiai et al., 2015a,b), followed by heart rate and heart rate variability (Song et al., 2013; Lee et al., 2014), cardiovascular disease-related pathological factors (Mao et al., 2012), salivary cortisol (Ochiai et al., 2015a), and urinary adrenaline and serum cortisol (Ochiai et al., 2015b). Psychologically, the mood states of participants, measured by Profile of Mood States (POMS) (Mao et al., 2012; Lee et al., 2014; Ochiai et al., 2015a,b), were by far the most focused aspect. Besides, subjective feelings that were evaluated by Semantic Differential (SD) method (Song et al., 2013; Lee et al., 2014; Ochiai et al., 2015b), and anxiety levels that were investigated by State-Trait Anxiety Inventory (STAI) (Song et al., 2013; Lee et al., 2014) were also frequently measured.

From 2016, the study contents became broader and more abundant, crossed with medicine, ecology, chemistry, and other disciplines. The most cited article in 2016 was the first systematic review with an evidence-based medical perspective (Song C. et al., 2016). The article elucidated the physiological effects of nature therapy and considered it an important preventive medicine in the future. Two controlled trials first found positive effects of forest therapy programs on people with chronic widespread pain (Han et al., 2016) and elderly patients with chronic obstructive pulmonary disease (Bing et al., 2016). Aerts et al. (2018) summarized the mechanisms of biodiversity within nature comprehensively and suggested specific attention to quantifying exposure to species diversity in further research. Kim et al. (2020) focused on the anti-inflammatory effects of forest aerosols and proposed using them as treatments for inflammatory-related diseases.

It is noteworthy that most of the high-impact articles mentioned above were written by researchers from Asian countries, mainly from Japan (Lee et al., 2014; Ochiai et al., 2015a,b; Song et al., 2013, 2015, Song C. et al., 2016), Korea (Song et al., 2013; Han et al., 2016; Kim et al., 2020), and China (Mao et al., 2012; Bing et al., 2016). But after 2016, interests in this field of study increased among researchers with different cultural backgrounds. For example, the American researchers Hansen et al. (2017) encouraged more longitudinal research conducted by healthcare professionals in Western cultures based on the clinical therapeutic effects of forest therapy research conducted in transcontinental Japan and China. Two field experiments on the evaluation of winter forest therapy effects (Bielinis et al., 2018) and the influences of different forest management decisions and/or ages on the restorative effects (Simkin et al., 2020) were conducted in Poland and Finland separately, which accumulated evidence in more diverse groups.

The top 10 citing articles in Cluster #1 was mostly new studies published between 2019 and 2021 (Supplementary Table 2), with a specific focus on the psychophysiological benefits of forests (1,3,5,6). Notably, forest bathing gets increasing attention from researchers from different countries, including Italy (1,3), Spain (5), and Hungary (6), while relevant studies are mainly conducted in the Asia-Pacific region (Peterfalvi et al., 2021).

#### Cluster #2

**FIGURE 10:**
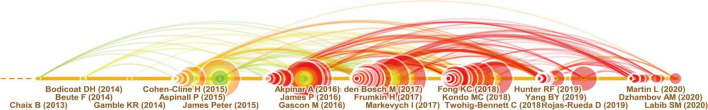
High-impact publications in Cluster #2.

Cluster #2 was the third-largest cluster with 119 references and was also one of the latest clusters with the median year of all references published in 2017 (Figure 10). Cluster #2 was labeled GIS and had a strong connection with the previous two clusters. According to the top 10 most cited articles (Supplementary Table 1), references in this cluster discussed the interlinks between environmental characteristics and health outcomes with the help of GIS to analyze the data spatially on a larger population scale.

Research in this cluster attached great importance to the measurements of greenspace exposure. Labib et al. (2020) mentioned that different evaluations of exposure to green-blue space may influence the strength of associations between greenness and research interests. James et al. (2015) conducted traditional reviews in 2015 and 2017 (Fong et al., 2018), respectively. It showed that the residential address remained the first choice for measuring exposure to surrounding levels of greenspace. But great heterogeneity was still found between studies regarding exposure assessment in the study by Gascon et al. (2016). This can be explained by different definitions of exposure, the use of various spatial datasets, and analysis methods, which varied in accordance with the research interests.

From the evidence-based experiments involving timeline visualization, we found continued efforts in improving the accuracy of results. First, the spatial resolution of data has gradually improved due to the greater availability of high-quality data, which has made it possible to capture small green spaces and avoid missing areas. The data varied from satellite images at a resolution of 25 m (Bodicoat et al., 2014), the National Land Cover Dataset with a 30 m × 30 m resolution (Akpinar et al., 2016), to the use of a Lower-layer Super Output Area at a resolution of 10 m (Martin et al., 2020). Second, as exposure misclassification may be caused by participants exercising in different places other than their residential address, some methods have been applied to capture personalized and dynamic data, such as location-tracking personal devices and time-weighted sums based on multiple important locations (Dadvand et al., 2017). Third, the spatially explicit measures of greenness became more comprehensive, from the single use of the Normalized Difference Vegetation Index (Cohen-Cline et al., 2015) to the National Land Cover Dataset (Akpinar et al., 2016), and the combined use of the Normalized Difference Vegetation Index and the Soil Adjusted Vegetation Index (Yang et al., 2019).

With the development of related disciplines, we noted that high-profile reviews have gradually begun to show multidisciplinary and international features. In 2017, the most cited article was a summary of pathways linking green space to health outcomes written by experts from transdisciplinary areas, including forestry, geography, remote sensing, environmental, and social epidemiology (Markevych et al., 2017). In 2019, researchers from America, Spain, and Switzerland were commissioned by the World Health Organization to analyze cohort studies that used the normalized difference vegetation index to estimate green space exposure. The research data covered more than eight million individuals from seven countries (Rojas-Rueda et al., 2019).

The top 10 citing articles (Supplementary Table 2) continually utilized GIS to explore the association between green space and geriatric depression (8) and all-cause mortality (10) on a large scale. One article was included in the above three clusters, summarizing the mechanisms of nature exposure on health benefits (Bratman et al., 2021). Besides, as health disparities have been a major health issue around the world (Rigolon et al., 2021), a new tendency in the top citing articles emerged, suggesting green space served as a tool to promote health equity (1, 2).

#### Remaining Clusters

As the timeline visualization shows, some clusters are no longer active. However, it does not indicate declining interest in these domains, but a shift from one relatively mature research area to another new hotspot. For example, although Cluster #3 (mental health), Cluster #4 (semantic differential method), and Cluster #6 (allostatic load) have no highly cited articles in recent years, articles in these clusters have been cited frequently by studies in other still active clusters as research basis. In addition, the relatively new research topics showed a trend toward the use of new technologies (Cluster #5 virtual reality) and the health co-benefits of human and natural systems (Cluster #7 public health, Cluster #8 planetary health).

## Discussion

This study first performed a bibliometric analysis of the worldwide research status of forest therapy and related issues from 2007 to 2021. Using WoSCC as a database, 2,545 publications were collected to investigate the knowledge structure and reveal the evolutionary process. From the visual networks, some information should be paid more attention.

### Does High Contribution Equal High Collaboration?

As shown in Table 2, we found that some countries had a high number of publications but without correspondingly high collaborative contributions. For example, China, Japan, and South Korea were the top 10 highest contributors to publications, but ranked only 30, 20, and 21, respectively, regarding centrality.

For China, we found the research topics of the top 10 most cited articles are relatively scattered (Supplementary Table 3), including forest therapy, ecosystem services, environmental epidemiology, and others. With the adoption of the “Healthy China 2030 plan,” the establishment of a forest therapy major in university, and the promulgation of policies on promoting population health (Tan et al., 2019; Yan et al., 2021), relevant literature has increased rapidly in recent years. But compared with earlier and more influential studies in The United States and England (Supplementary Table 3), Chinese research published later had less chance to be cited as the new manuscripts are more likely to cite the most cited manuscripts (Chu and Evans, 2021). Most of the top 10 cited articles from Japan and South Korea were empirical studies conducted on less than 200 participants to explore the effects of forest bathing (Supplementary Table 3). The low centrality may be related to insufficient practice under Western culture and relatively low generalization due to the limitation of geography and small sample size.

The above problems bring us the following inspiration: first, for researchers new to this area, more focus on the abovementioned important topics is needed to get an in-depth understanding of the worldwide issues to promote the development of the discipline. Second, since individual environmental preferences and attitudes can be influenced by culture (Kountouris and Remoundou, 2016; Xia et al., 2021), it is necessary to conduct experiments in different cultural backgrounds with larger sample sizes to obtain more general results. Third, international cooperation can be enhanced by the use of novel methods. For example, the utilization of VR can break down the spatial and temporal barriers so as to increase statistical power and further ensure research validity (Reese et al., 2022).

### Stepped Toward Preventive Medicine?

More than half of the high-impact contributions in the timeline visualization were reviews, including traditional reviews, systemic reviews, and meta-analysis. Accumulated research provided positive evidence of nature-based therapy for the prevention and treatment of mental health problems, reduction of stress hormone levels, health regulating effects of green space, etc. (Dzhambov et al., 2020; Park et al., 2022).

However, most of the primary studies had quality problems or risk of bias, including study design, research process, report quality, and so on (Stier-Jarmer et al., 2021; Yeon et al., 2021). Therefore, more detailed and rigorous research is needed in future evidence-based experiments, such as using strict randomized controlled trials and blinded design of experiments, adopting objective physiological indicators (e.g., electroencephalograph and facial action coding system) to assist in confirming subjective psychological outcomes, and focusing on the dose of green space exposure to obtain the maximum effects on health and its durations (Labib et al., 2020; Yeon et al., 2021).

The progressive growth of systemic reviews and meta-analyses suggested that the research showed a tendency from early empirical knowledge to potential non-pharmacological treatments, which will help nature therapy serve as preventive medicine to reduce medical expenditures of the whole society (Yeon et al., 2021; Park et al., 2022). However, it is noteworthy that the systematic errors can affect the accuracy of the results of meta-analyses, such as different search strategies, selection criteria, research heterogeneity, and misestimated effect sizes caused by a small number of primary studies (Mikolajewicz and Komarova, 2019; Rojas-Rueda et al., 2019; Rosa et al., 2021; Stier-Jarmer et al., 2021; Yeon et al., 2021; Park et al., 2022). Accordingly, future meta-analyses should pay attention to the comprehensiveness of the publication language and time in search strategies, the quality of selected articles, and more cautious interpretation of the results (Mikolajewicz and Komarova, 2019; Rojas-Rueda et al., 2019; Rosa et al., 2021; Stier-Jarmer et al., 2021; Yeon et al., 2021; Park et al., 2022).

### Not Only Forest Therapy?

Major clusters classified by reference co-citation reflected the knowledge structure and developments of forest therapy research, which can be further categorized into two groups, namely the relationship between humans and nature and relevant research tools. As shown in Figure 7, the research hotspots evolved from the early focus on individual benefits (#3 mental health; #6 allostatic load) to the dynamic interactions between nature and humans (#0 green space; #1 forest therapy), and further to increasing attention on human well-being at social-ecological scale (#7 public health; #8 planetary health), also consistent with the results of burst citations. The tendency was related to the knowledge fundaments of forest therapy in great multiple disciplines, from environmental sciences to psychology and public health (Supplementary Figure 1). The academic understanding of relationships between nature and humans changed dynamically in accordance with the development of relational epistemologies. For example, the research approach shifted from the traditional western human–nature dichotomy, which was seen as a barrier to sustainable development and ecological well-being (Caillon et al., 2017; Welden et al., 2021), to discussing human health from a systems-oriented perspective, such as planetary health – a more recent concept proposed by The Rockefeller Foundation-Lancet Commission as “the highest attainable standard of health, well-being, and equity worldwide” (Whitmee et al., 2015) and also one of the newest clusters in our researches.

However, we found a deficiency of input from social disciplines, which may limit the better understanding of causal effects and further translation from scientific evidence to policy making. According to the “doughnut model,” the human well-being in the Anthropocene (Waters et al., 2016) needed to consider both social foundation and ecological ceiling to guide ecosystem sustainable development (Raworth, 2017). Therefore, more interdisciplinary collaboration among sociologists, psychologists, ecologists, and medical scientists is needed to address increasingly complex scenarios from a more holistic perspective and to communicate evidence-based recommendations to the public and governments following a comprehensive assessment of multilevel consequences of behaviors and policies, including the benefits and harms to people and the planet (Myers, 2017; Iyer et al., 2021).

From the analysis results, we assumed that the forest therapy and related research have entered the third stage of the scientific research process in Shneider’s four-stage theory (Shneider, 2009), where the current research has previously been biased toward the application of new tools for solving new problems after the creation of a new discipline and the development of new methods. Future researchers could, on the one hand, utilize new techniques to broaden the research scope, such as facial recognition software, wearable technologies, and immersive virtual environment techniques (Liu P. et al., 2021; Fu et al., 2022; Jiang et al., 2022). On the other hand, analytic approaches to assessing the impacts of policy decisions on nature are also noteworthy, especially since they had been successfully applied as decision-making tools for introducing health into policies. For example, tools like the Health Impact Assessment, the Environmental Benefits Mapping and Analysis Program – Community Edition, the “doughnut model”, and others will continue playing an important role in making better transport planning, quantifying the human health impacts of air quality, conducting global cross-national analysis, and so on (Sacks et al., 2018; Khomenko et al., 2020; Fanning et al., 2022).

## Conclusion

In this study, a bibliometric analysis was conducted to investigate the intellectual structure of forest therapy-related studies for the period from 2007 to 2021. The increasing number of publications demonstrated that the research on forest therapy has gained growing worldwide interest. Environmental sciences and ecology were the fields that contributed most and the *International Journal of Environmental Research and Public Health* had the most studies published, but the input from social fields was relatively lacking. Regarding countries involved in relevant research, the United States of America contributed the most publications while some Asian countries with high study numbers but relatively low centralities. The results of citation bursts showed the research hotspots varied at different times, from the focus on individual health to human well-being in a healthy earth system. As shown in cluster analysis, the knowledge fundaments of forest therapy are not limited to forest context, but include various topics across numerous disciplines, with green space, forest therapy, and GIS ranked the top three largest clusters.

Forest therapy research is in the third stage of the scientific research process, where more studies using novel technologies and decision-making frameworks to solve practical issues will emerge and further provide scientific evidence to the governments and the public. More rigorous experiments are needed for future studies with strict randomized controlled trials, blinding, and the combination of objective and subjective metrics. Meanwhile, international and interdisciplinary collaboration is also needed for future studies to view complex systems from multilevel perspectives and consider the health of both humans and the ecosystem. Compared with other reviews, this study provides researchers with a holistic and in-depth understanding of the worldwide research hotspots and their evolution tendency of forest therapy-related research in an intuitive and objective way.

This study has some limitations as follows: first, considering that data retrieved from other databases must be transformed to accommodate the use of CiteSpace, we chose WoSCC as the final data source in order to avoid the loss of key information for each publication. However, this may result in an incomplete collection of relevant articles from other databases. Second, all of the data in this study were derived from English articles while the inclusion of articles written in other languages may have yielded more innovative results.

## Data Availability Statement

The original contributions presented in the study are included in the article, further inquiries can be directed to the corresponding author.

## Author Contributions

XW and W-YC: conceptualization. K-XX, D-SG, and L-JL: methodology. XW and X-FG: software and visualization. XW, X-FG, K-XX, and D-SG: writing—original draft preparation. XW, L-JL, C-ML, and W-YC: writing—review and editing. All authors have read and agreed to the published version of the manuscript.

## Conflict of Interest

The authors declare that the research was conducted in the absence of any commercial or financial relationships that could be construed as a potential conflict of interest.

## Publisher’s Note

All claims expressed in this article are solely those of the authors and do not necessarily represent those of their affiliated organizations, or those of the publisher, the editors and the reviewers. Any product that may be evaluated in this article, or claim that may be made by its manufacturer, is not guaranteed or endorsed by the publisher.
