# Supplementary material for: Mapping of Research in the Field of Forest Therapy-Related Issues: A Bibliometric Analysis for 2007–2021
Source: Front Psychol. 2022 Jul 11;13:930713. doi: 10.3389/fpsyg.2022.930713 (PMC9309728; doi:10.3389/fpsyg.2022.930713)
Supplement: Supplementary file 1 [file Data_Sheet_1.pdf]

Supplementary Material

1 Supplementary Figures

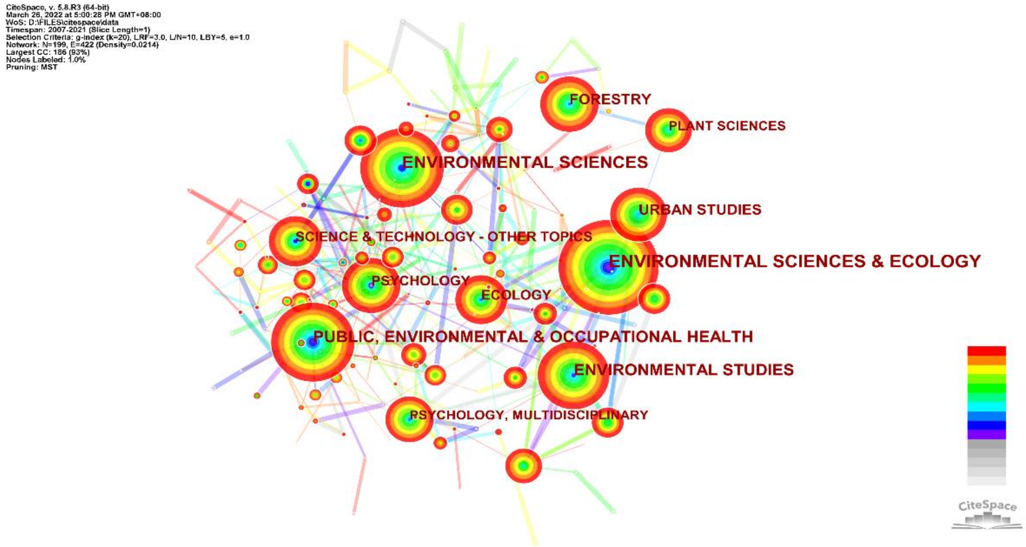

Fig. S1. Subject Categories of publications.

2 Supplementary Tables

Table S1. The top 10 most cited references of Cluster# 0 - 2.

Clusters	Frequency	Author	Year	Title
0	285	Sandifer, Paul A. et al.	2015	Exploring connections among nature, biodiversity, ecosystem services, and human health and well-being: Opportunities to enhance health and biodiversity conservation
0	165	Hartig T. et al.	2014	Nature and Health
0	107	McMahan EA. et al.	2015	The effect of contact with natural environments on positive and negative affect: A meta-analysis
0	92	Shanahan DF. et al.	2016	Health Benefits from Nature Experiences Depend on Dose
0	72	Bratman GN. et al.	2015	Nature experience reduces rumination and subgenual prefrontal cortex activation
0	72	Heather Ohly et al.	2016	Attention Restoration Theory: A systematic review of the attention restoration potential of exposure to natural environments
0	72	Gascon M. et al.	2015	Mental Health Benefits of Long-Term Exposure to Residential Green and Blue Spaces: A Systematic Review
0	69	Bratman GN. et al.	2015	The benefits of nature experience: Improved affect and cognition

0	67	Carrus G. et al.	2015	Go greener, feel better? The positive effects of biodiversity on the well-being of individuals visiting, urban and peri-urban green areas
0	67	Kuo M. et al.	2015	How might contact with nature promote human health? Promising mechanisms and a possible central pathway
1	115	Hansen MM. et al.	2017	Shinrin-Yoku (Forest Bathing) and Nature Therapy: A State-of-the-Art Review
1	93	Song CR. et al.	2016	Physiological Effects of Nature Therapy: A Review of the Research in Japan
1	60	Ochiai H. et al.	2015	Physiological and Psychological Effects of Forest Therapy on Middle-Aged Males with High-Normal Blood Pressure
1	59	Lee J. et al.	2014	Influence of Forest Therapy on Cardiovascular Relaxation in Young Adults
1	56	Bielinis E. et al.	2018	The effect of winter forest bathing on psychological relaxation of young Polish adults
1	52	Ochiai H. et al.	2015	Physiological and Psychological Effects of a Forest Therapy Program on Middle-Aged Females
1	52	Lee I. et al.	2017	Effects of Forest Therapy on Depressive Symptoms among Adults: A Systematic Review
1	51	Song CR. et al.	2015	Physiological and Psychological Effects of a Walk in Urban Parks in Fall
1	50	Yu CP. et al.	2017	Effects of Short Forest Bathing Program on Autonomic Nervous System Activity and Mood States in Middle-Aged and Elderly Individuals
1	46	Chun MH. et al.	2017	The effects of forest therapy on depression and anxiety in patients with chronic stroke
2	163	Markevych I. et al.	2017	Exploring pathways linking greenspace to health: Theoretical and methodological guidance
2	138	Twohig-Bennett C. et al.	2018	The health benefits of the great outdoors: A systematic review and meta-analysis of greenspace exposure and health outcomes
2	111	Frumkin H. et al.	2017	Nature Contact and Human Health: A Research Agenda
2	87	Kondo MC. et al.	2018	Urban Green Space and Its Impact on Human Health
2	73	Gascon M. et al.	2016	Residential green spaces and mortality: A systematic review
2	72	Rojas-Rueda D. et al.	2019	Green spaces and mortality: a systematic review and meta-analysis of cohort studies
2	71	James P. et al.	2016	Exposure to Greenness and Mortality in a Nationwide Prospective Cohort Study of Women
2	68	James Peter et al.	2015	A Review of the Health Benefits of Greenness
2	67	Van den Bosch M. et al.	2017	Urban natural environments as nature-based solutions for improved public health - A systematic review of reviews
2	66	Akpinar A. et al.	2016	Does green space matter? Exploring relationships between green space type and health indicators

Table S2. The top 10 citing references of Cluster# 0 - 2.

Cluster	Rank	Coverage	Author	Year	Title
0	1	47	Bratman et al.	2019	Nature and mental health: An ecosystem service perspective
0	2	46	Frumkin et al.	2017	Nature Contact and Human Health: A Research Agenda
0	3	43	Wendelboe-Nelson et al.	2019	A Scoping Review Mapping Research on Green Space and Associated Mental Health Benefits
0	4	32	Bratman et al.	2021	The affective benefits of nature exposure
0	5	31	Roberts et al.	2019	The effect of short-term exposure to the natural environment on depressive mood: A systematic review and meta-analysis
0	6	30	Buckley et al.	2017	Economic Value of Parks via Human Mental Health: An Analytical Framework
0	7	27	Aerts et al.	2018	Biodiversity and human health: mechanisms and evidence of the positive health effects of diversity in nature and green spaces
0	8	27	Kondo et al.	2018	Urban Green Space and Its Impact on Human Health
0	9	27	Jimenez et al.	2021	Associations between Nature Exposure and Health: A Review of the Evidence
0	10	26	Reeves et al.	2021	A Qualitative Analysis of UK Wetland Visitor Centres as a Health Resource
1	1	41	Doimo et al.	2020	Forest and Well-being: Bridging Medical and Forest Research for Effective Forest-Based Initiatives
1	2	33	Logan et al.	2015	Natural environments, ancestral diets, and microbial ecology: is there a modern "paleo-deficit disorder"? Part I
1	3	30	Antonelli et al.	2021	Effects of forest bathing (shinrin-yoku) on individual well-being: an umbrella review
1	4	27	Bratman et al.	2021	The affective benefits of nature exposure
1	5	25	Bach Pages et al.	2020	How Should Forests Be Characterized in Regard to Human Health? Evidence from Existing Literature
1	6	25	Peterfalvi et al.	2021	Forest Bathing Always Makes Sense: Blood Pressure-Lowering and Immune System-Balancing Effects in Late Spring and Winter in Central Europe
1	7	23	Wendelboe-Nelson et al.	2019	A Scoping Review Mapping Research on Green Space and Associated Mental Health Benefits
1	8	23	Kanelli et al.	2021	Engaging the Senses: The Association of Urban Green Space with General Health and Well-Being in Urban Residents
1	9	22	Wilkie et al.	2021	Prevalence and effectiveness of nature-based interventions to impact adult health-related behaviors and outcomes: a scoping review
1	10	22	Rahimi-Ardabili et al.	2021	Green Space and Health in Mainland China: A Systematic Review
2	1	31	Rigolon et al.	2021	Green Space and Health Equity: A Systematic Review on the Potential of Green Space to Reduce Health Disparities

2	2	26	Bratman et al.	2019	Nature and mental health: An ecosystem service perspective
2	3	23	Bratman et al.	2021	The affective benefits of nature exposure (Bratman et al., 2021b)
2	4	22	Dzhambov et al.	2020	Analytical approaches to testing pathways linking greenspace to health: A scoping review of the empirical literature
2	5	22	Reeves et al.	2021	A Qualitative Analysis of UK Wetland Visitor Centres as a Health Resource
2	6	21	Marselle et al.	2021	Biodiversity and Health in the Urban Environment
2	7	21	Labib et al.	2010	Scale effects in remotely sensed greenspace metrics and how to mitigate them for environmental health exposure assessment
2	8	19	Peng et al.	2021	Association of residential greenness with geriatric depression among the elderly covered by long-term care insurance in Shanghai
2	9	18	Liu et al.	2021	Natural outdoor environments and subjective well-being in Guangzhou, China: Comparing different measures of access
2	10	18	Kua et al.	2021	The influence of residential greenness on mortality in the AsiaPacific region: a systematic review and meta-analysis

Table S3. The most citied articles in the high contributed countries.

Countries	N/C	Citations	Year	Title
USA	1/2	682	2012	Contributions of cultural services to the ecosystem services agenda
USA	1/2	436	2015	Exploring connections among nature, biodiversity, ecosystem services, and human health and well-being: Opportunities to enhance health and biodiversity conservation
USA	1/2	420	2012	The impacts of nature experience on human cognitive function and mental health
USA	1/2	356	2007	Determinants of the diurnal course of salivary alpha-amylase
USA	1/2	351	2009	Determinants of salivary alpha-amylase in humans and methodological considerations
USA	1/2	349	2012	Biodiversity and the Feel-Good Factor: Understanding Associations between Self-Reported Human Well-being and Species Richness
USA	1/2	315	2017	Nature Contact and Human Health: A Research Agenda
USA	1/2	296	2011	A Difference-in-Differences Analysis of Health, Safety, and Greening Vacant Urban Space
USA	1/2	295	2017	Biodiversity in the city: key challenges for urban green space management
USA	1/2	285	2014	Exposure to Neighborhood Green Space and Mental Health: Evidence from the Survey of the Health of Wisconsin
England	2/1	682	2012	Contributions of cultural services to the ecosystem services agenda

England	2/1	545	2011	Does Participating in Physical Activity in Outdoor Natural Environments Have a Greater Effect on Physical and Mental Well-being than Physical Activity Indoors? A Systematic Review
England	2/1	515	2012	More green space is linked to less stress in deprived communities: Evidence from salivary cortisol patterns
England	2/1	442	2013	What are the Benefits of Interacting with Nature?
England	2/1	402	2016	Has land use pushed terrestrial biodiversity beyond the planetary boundary? A global assessment
England	2/1	349	2012	Biodiversity and the Feel-Good Factor: Understanding Associations between Self-Reported Human Well-being and Species Richness SO BIOSCIENCE
England	2/1	337	2018	The health benefits of the great outdoors: A systematic review and meta-analysis of greenspace exposure and health outcomes
England	2/1	295	2017	Biodiversity in the city: key challenges for urban green space management
England	2/1	257	2013	Green Space and Stress: Evidence from Cortisol Measures in Deprived Urban Communities
England	2/1	233	2015	Human-environment interactions in urban green spaces - A systematic review of contemporary issues and prospects for future research
Australia	4/3	442	2013	What are the Benefits of Interacting with Nature?
Australia	4/3	402	2016	Has land use pushed terrestrial biodiversity beyond the planetary boundary? A global assessment
Australia	4/3	389	2008	Associations of neighbourhood greenness with physical and mental health: do walking, social coherence and local social interaction explain the relationships?
Australia	4/3	170	2015	The Health Benefits of Urban Nature: How Much Do We Need?
Australia	4/3	147	2013	Relationships between place attachment, place satisfaction and pro-environmental behaviour in an Australian national park
Australia	4/3	147	2014	Evaluating restoration in urban green spaces: Does setting type make a difference?
Australia	4/3	131	2015	40-second green roof views sustain attention: The role of micro-breaks in attention restoration
Australia	4/3	128	2017	Doses of Neighborhood Nature: The Benefits for Mental Health of Living with Nature
Australia	4/3	128	2009	Associations Between Neighborhood Open Space Attributes and Quality of Life for Older People in Britain
Australia	4/3	102	2018	Climate change and mental health: risks, impacts and priority actions
China	2/30	233	2019	Nature and mental health: An ecosystem service perspective
China	2/30	159	2014	A dose of nature: Tree cover, stress reduction, and gender differences
China	2/30	105	2018	Effects of urban form on the urban heat island effect based on spatial regression model

China	2/30	90	2012	Therapeutic effect of forest bathing on human hypertension in the elderly
China	2/30	82	2018	Residential greenness and prevalence of major depressive disorders: a cross-sectional, observational, associational study of 94 879 adult
China	2/30	78	2019	Impacts of urbanization-induced land-use changes on ecosystem services: A case study of the Pearl River Delta Metropolitan Region, China
China	2/30	75	2019	Exploring the disparities in park access through mobile phone data: Evidence from Shanghai, China
China	2/30	69	2012	Effects of Short-Term Forest Bathing on Human Health in a Broad-Leaved Evergreen Forest in Zhejiang Province, China
China	2/30	68	2015	A dose-response curve describing the relationship between tree cover density and landscape preference
China	2/30	67	2016	A Dose-Response Curve Describing the Relationship Between Urban Tree Cover Density and Self-Reported Stress Recovery
Japan	5/20	386	2014	The influence of urban green environments on stress relief measures: A field experiment
Japan	5/20	240	2011	Effect of forest bathing on physiological and psychological responses in young Japanese male subjects
Japan	5/20	188	2013	Physiological and psychological effects of viewing urban forest landscapes assessed by multiple measurements
Japan	5/20	152	2008	Visiting a forest, but not a city, increases human natural killer activity and expression of anti-cancer proteins
Japan	5/20	145	2009	Restorative effects of viewing real forest landscapes, based on a comparison with urban landscapes
Japan	5/20	131	2008	A forest bathing trip increases human natural killer activity and expression of anti-cancer proteins in female subjects
Japan	5/20	124	2009	Physiological Effects of Forest Recreation in a Young Conifer Forest in Hinokage Town
Japan	5/20	124	2011	Relationship between psychological responses and physical environments in forest settings
Japan	5/20	119	2014	Influence of Forest Therapy on Cardiovascular Relaxation in Young Adults
Japan	5/20	112	2016	Physiological Effects of Nature Therapy: A Review of the Research in Japan
South Korea	6/21	240	2011	Effect of forest bathing on physiological and psychological responses in young Japanese male subjects
South Korea	6/21	188	2013	Physiological and psychological effects of viewing urban forest landscapes assessed by multiple measurements
South Korea	6/21	124	2011	Relationship between psychological responses and physical environments in forest settings
South Korea	6/21	119	2014	Influence of Forest Therapy on Cardiovascular Relaxation in Young Adults
South Korea	6/21	100	2014	Emotional, Restorative and Vitalizing Effects of Forest and Urban Environments at Four Sites in Japan

South Korea	6/21	81	2012	The Effect of Cognitive Behavior Therapy-Based "Forest Therapy" Program on Blood Pressure, Salivary Cortisol Level, and Quality of Life in Elderly Hypertensive Patients
South Korea	6/21	77	2013	Physiological and psychological effects of walking on young males in urban parks in winter
South Korea	6/21	55	2017	Effects of Forest Therapy on Depressive Symptoms among Adults: A Systematic Review
South Korea	6/21	"53	2017	Health and well-being benefits of spending time in forests: systematic review
South Korea	6/21	55	2014	Browning control of fresh-cut lettuce by phytoncide treatment

Note: USA: the United States of America; N/C: Rank of the number of publications/ Rank of the centralities
